# Better prognostic marker in ICU - APACHE II, SOFA or SAP II!

**DOI:** 10.12669/pjms.325.10080

**Published:** 2016

**Authors:** Iftikhar Haider Naqvi, Khalid Mahmood, Syed Ziaullaha, Syed Mohammad Kashif, Asim Sharif

**Affiliations:** 1 Dr. Iftikhar Haider Naqvi, MBBS, FCPS. Department of Medicine, Dow University of Health Sciences, Karachi, Pakistan; 2 Prof. Khalid Mahmood, MBBS, FCPS, FRCP(E), FRCP(G). Department of Medicine, Dow University of Health Sciences, Karachi, Pakistan; 3 Dr. Syed Ziaullah, MBBS, FCPS II Trainee. Department of Medicine, Dow University of Health Sciences, Karachi, Pakistan; 4 Dr. Syed Muhammad Kashif, MBBS, FCPS. Department of Medicine, Dow University of Health Sciences, Karachi, Pakistan; 5 Dr. Asim Sharif, MBBS, FCPS II Trainee. Department of Medicine, Dow University of Health Sciences, Karachi, Pakistan

**Keywords:** Critically ill patients, ICU mortality, Acute Physiology and Chronic Health Evaluation (APACHE II), Simplified Acute Physiology Score (SAPS II). SOFA: Sequential Organ Failure Assessment

## Abstract

**Objectives:**

This study was designed to determine the comparative efficacy of different scoring system in assessing the prognosis of critically ill patients.

**Methods:**

This was a retrospective study conducted in medical intensive care unit (MICU) and high dependency unit (HDU) Medical Unit III, Civil Hospital, from April 2012 to August 2012. All patients over age 16 years old who have fulfilled the criteria for MICU admission were included. Predictive mortality of APACHE II, SAP II and SOFA were calculated. Calibration and discrimination were used for validity of each scoring model.

**Results:**

A total of 96 patients with equal gender distribution were enrolled. The average APACHE II score in non-survivors (27.97+8.53) was higher than survivors (15.82+8.79) with statistically significant p value (<0.001). The average SOFA score in non-survivors (9.68+4.88) was higher than survivors (5.63+3.63) with statistically significant p value (<0.001). SAP II average score in non-survivors (53.71+19.05) was higher than survivors (30.18+16.24) with statistically significant p value (<0.001).

**Conclusion:**

All three tested scoring models (APACHE II, SAP II and SOFA) would be accurate enough for a general description of our ICU patients. APACHE II has showed better calibration and discrimination power than SAP II and SOFA.

## INTRODUCTION

Intensive care units (ICU) in most settings consume very high cost and sophisticated devices but mortality rates are still very high. There has been a great advancement recently in developing various models to measure severity of critically ill patients and to predict their mortality. Several models like APACHE II, SAP and SOFA have been devised for mortality prediction in critical ill patients.^1^ The evaluation of quality of intensive care can be effectively determined only by those scoring model which quantify the severity of illness.^2^-^5^ A perfect scoring model to predict outcome requires, precise data on severity of illness with associated risk of death. However for any scoring system and its related risk, prediction model is considered useful only if it demonstrates both good discrimination and calibration.^6^,^7^

These indices not only provide the assessment of various ICU performances but also give cost effectiveness of these services. APACHE II (acute physiology and chronic health evaluation II)and the SAPS II (simplified acute physiology score II) are commonly used scoring system for severity of illness in intensive care.^7^-^9^ APACHE II and SAPII were developed for the general ICU population and can predict the risk of in-hospital death.^8^,^9^ SOFA (sequential organ failure assessment) is another commonly used scoring system which is related to organ failure and used for prediction of outcome.^10^-^15^ APACHE II is the most broadly used model where only 12 physiological variables were included. This model has incorporated chronicity of health and effects of age influenced according to their relative impact. It can give a single score with a maximum of 71. APACHE II is applied within 24 hours of ICU admission with worst value recorded for each component part of physiology variable. The principal diagnosis responsible to ICU admission is put in APACHE II as a category so as to observe the predicted mortality based on principal diagnosis at admission.^16^ APACHE II score of 25 correspond to a predicted mortality of 50% and a score >35 signifies a predicted mortality of 80%. SAPS used 13 weighted physiological variables and age to predict risk of death in ICU patients. SAPS are applied within first 24 hours of ICU admission where worst values were recorded. The new SAPS II^17^ has total 17 variables which include 12 physiological variables, admission type, age and underlying disease related variables (3). The Sequential Organ Failure Assessment (SOFA) is an objective scoring model to offer an improved stratification of the mortality risk in ICU. This model uses the severity of organ dysfunction in terms of numbers of six organ system of body including Liver, lungs, coagulatory, CVS, renal, and neurologic (each 1–4) to offer a final score [6–24 (maximum)]. SOFA score computes individual or cumulative organ dysfunction. SOFA score is calculated at the time of admission and subsequently every 24 hours till discharge.^18^ Most of these scoring systems except SOFA and mortality prediction model (MPM) have good sensitivity and specificity if applied during first 24 hours of admission in ICU.

By applying logistic regression the APACHE II and SAPS II systems calculate the individual risk of hospital death by changing the score into probability of death. Acute Physiology and Chronic Health Evaluation (APACHE) II and Simplified Acute Physiology Score (SAPS) II assess severity of illness on physiologic variables in the form of numeric score. Higher score of these models indicate more severity of illness due to their impact on mortality. The numeric scores of APACHE II and SAP II are usually converted into predicted mortality with a logistic regression formula designed and validated on ICU patients.

The application and comparison of various scoring system like APACH II, SAP and SOFA has been limited in the Pakistani ICUs. This study was designed for a public sector hospital with immense burden of patients in critical state having admitted to ICU so as to know the comparative efficacy of different scoring system for prognosis assessment.

## METHODS

This was a retrospective study conducted in medical intensive care unit (MICU) and high dependency unit (HDU) medical unit III, Civil Hospital, a largest public tertiary care center in Karachi from April 2012 to August 2012. Out of 123 patients over 16 years of age who fulfilled the criteria of MICU admission only 96 cases with complete information about APACHE II, SAPS II and SOFA scores in case record were finally enrolled for the study. All enrolled patients were followed until their discharge from ICU and HDU or death and their discharge from the hospital. Twenty nine patients with incomplete information of scoring system in case records were labeled as missing data. All other patients including coronary care patients, patients admitted for observation and patients with readmission were excluded. After admission to the ICU, APACHE II, SAPS II, and sofa were calculated in accordance with the original methodology, using the worst physiological values on the first ICU day. During further treatment in the ICU, SOFA was calculated at 24 hour, 48 hour, 72 hour, and 7 days after admission using certain laboratory and radiological variables.

The variable of APACHE II, SAPS II and SOFA scores as specified were used to arrange the formal research instrument. The author himself collected all relevant data including demographic profile, reason for ICU admission, presence of chronic disease, prior history of hospitalization, ICU admission and severity of illness. Total length of intensive care and hospital stay were also recorded. All data was retrieved from comprehensive chart used for patients admitted in ICU. For survival status patients were followed till ICU and hospital discharge.

### Statistical analysis

Data were analyzed by Statistical Package for Social Sciences (SPSS, version 16.0; SPSS Inc., Chicago, IL) for Windows. Predictive mortality of APACHE II, SAP II and SOFA were calculated. Data were expressed as mean ± SD and frequencies as appropriate. Chi square and student t-test of statistical significance were applied for categorical and continuous variable respectively where p value of < 0.05 was considered to be statistically significant. Calibration and discrimination was used for validity of each scoring model.

### Calibration

Calibration defined as the degree of correspondence between predicted and observed mortality over the whole range of risks, was assessed by Hosmer-Lemeshow goodness to fit C statistic. As a matter of fact, a model with lower Hosmer-Lemeshow value and higher P value>0.05 was considered better.

### Discrimination

Discrimination defined as the model’s ability to differentiate between patients who died and those who survived, was assessed by receiver operation characteristic (ROC) curves. A model with greater AUC (area under curve) was considered better. Finally a cut off value was calculated, sensitivity, specificity, overall correctness of prediction was determined and comparison among survivors and non-survivors was done using odds ratio.

## RESULTS

**Fig.1:**
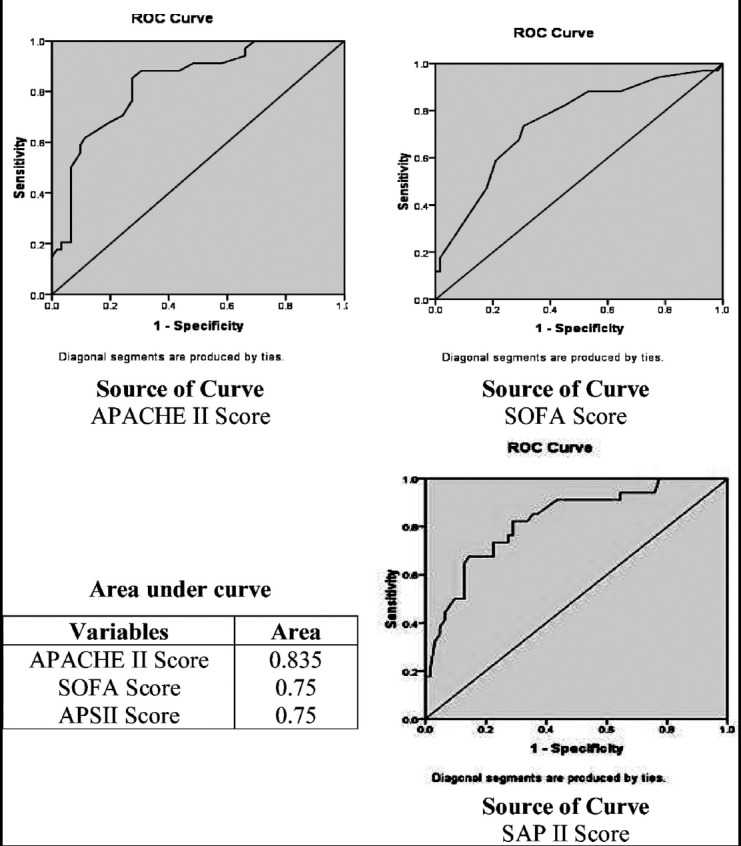
ROC curves of APACHE II, SOFA and SAPII on prediction of mortality.

A total of 96 patients with equal gender distribution were included in the study. Patients were meanly aged 32.93±16.61 years. The most common diagnosis was organophosphate poisoning for the ICU admission 28(29.2%), followed by septicemia 10(10.4%) and others. The mean length of stay in ICU was 9.06±11.97days, while mean length of stay in ward was 3.04±5.20 days. Out of total patients 62(64.6%) were discharged to ward first and then eventually to home after their complete recovery and 34 (35.4%) died. in this study. Most of patients who died had hepatic encephalopathy (41.1%) and septicemia (32.3%) followed by pulmonary embolism (8.8%), organophosphate poisoning (5.8%), fulminant hepatic failure (5.8%), DKA (2.9%) and stroke (2.9%). Demographic profile, score of APACHE II, SAP II, and SOFA along with their predicted mortality is given in Table-I. The average APACHE II score in non-survivors (27.97±8.53) was higher than survivors (15.82±8.79) with statistically significant p value (<.001). The average SOFA score in non-survivors (9.68±4.88) was higher than survivors (5.63±3.63) with statistically significant p value (<.001). SAP II average score in non-survivors (53.71±19.05) was higher than survivors (30.18±16.24) with statistically significant p value (<.001). Comparison of various models among survivors and non survivors is shown in Table-II. Calibration of each scoring system exhibited good effectiveness. The goodness of fit Hosmer-Lemeshow test and p value of each scoring system is shown in Table-III. This shows that APACHEII performed better in our ICU & HDU. The overall discriminative capability as determined by ROC curve is shown in Fig.1

**Table-I:** Demographic profile and characteristics of ICU patients.

*Demographic variables*	*Mean ± SD*
Age	32.93±16.61
ICU mortality	35.4
length of stay in ICU	9.06±11.97
*Severity assessment models*	
APACHE II score	20.1± 10.44
SAP II score	38.51±20.57
SOFA score	7.06± 4.73
*Predicted mortality*	
APACHE II	39.82±27.54
SAPSII	1.96±1.36
SOFA	30.50±29.6

**Table-II:** Comparison of survivors versus non survivors.

*Scoring Model*	*Survivors*	*Non Survivors*	*p-value*
APACHE II	15.82±8.79	27.97±8.53	<.001
SOFA	5.63±3.63	9.68±4.88	<.001
SAPS II	30.18±16.24	53.71±19.05	<.001

**Table-III:** Goodness of fit Hosmer-Lemeshow test and p value of each scoring model.

*Scoring Model*	*Chi square*	*P-value*
APACHE II	3.199	0.866
SOFA	7.679	0.362
SAPS II	3.724	0.811

## DISCUSSION

Current study evaluated the capability and validity of three ICU scoring models (APACHE II, SOFA and SAPS II) to predict accurately the mortality in an ICU. All three models demonstrated good calibration and discrimination. APACHE II showed better performance on Intermodel comparison as compare to SOFA and SAP II. The patients in this study were young (32.93±16.61) as compare to earlier studies where mean ages were (61.06 ± 15.42 years, 63.9 ± 17.6 years) respectively.^19^,^20^ The young aged patients in this study could be owing to organophosphate poisoning, the commonest reason for admission.

The mean ICU time-span was 9.06±11.97days which is in agreement to the earlier studies.^19^,^20^ The mortality rate in this current study was 35.4% which is concurrent to earlier published studies.^19^,^21^ The Freire P et al. and Mbongo CU et al. in their studies had different results and showed low ICU mortality 8.2% and 5.3% respectively.^20^,^22^ The mean APACHE II score in this study is 20.1± 10.44 with its predicted mortality of 39.82±27.54 which is in accordance to earlier studies.^22^,^19^ Mean SOFA & SAP II scores were 38.51±20.57 and 7.06± 4.73 with predicted mortality of 1.96±1.36,1.96±1.36 respectively, similar to earlier published studies.^19^,^23^ The above referred studies were also done on similar kind of patients in medical ICU of Bangladesh and Iran where readmissions to ICU and patients with coronary care were also excluded like our study. When compared between survivors and non survivors this study showed higher scores of all three models (APACHE II, SAP II, SOFA) in non survivors than survivors with statistically significant p value of <0.001. Knaus et al.^19^ has also shown the similar results among survivors versus nonsurvivors. APACHE II, SAPS II and SOFA models for prediction of mortality in this study showed good effectiveness when tested on calibration, although APACHE II showed slightly better effectiveness than SAP II and SOFA in ICU/HDU because of lower Hosmer-Lemeshow value and higher P value>0.05 as compare to SAPS II and SOFA. Each model in this current study showed good discriminative power as assessed by area under the ROC (Receiver operator curve) while APACHE II showed better discriminative power than SAP II and SOFA due to its greater value(.83) of area under ROC (Receiver operator curve) as compare to .75 for both SAPS II and SOFA. ICU mortality prediction studies^25^-^29^ published earlier have reported good discrimination among scoring models like this study.

Prediction of mortality by various models is influenced by various factors like highest and lowest scoring value and GCS level in APACHE II. The indecisiveness of GCS determination in sedated patients might affect the predicted death in all models. This study uses pre-sedation GCS determination in sedated patients like previous studies.^26^,^29^ Calibration can be inaccurate if different medical definitions and inclusion criteria are used in the databases. The above problem is rectified in current study by using standard medical definitions from original publications. Higher predictive mortality in our ICU setting as compared to western studies by using same scoring model indicates less good quality of ICU in developed countries. Accuracy of risk prediction can also affect by lead time bias. Tunnell et al.^30^ in their study showed that lead time bias amplified the APACHE II and SAPSII scores by 14 and 23 points, respectively, which ultimately increased the APACHE II and SAPS II for prediction of hospital mortality as much as 42.7% and 33.4%, respectively. Partial treatment offered to patient before ICU admission causes Lead time bias which is responsible for underestimation of the severity of underlying disease. The quantification of lead time bias is difficult in this study but its effect is narrow due to limited Intensive care facility where most of patients admitted to the emergency department were shifted to the ICU without significant vital support.

### Limitations of the study

Firstly the study design is retrospective and secondly all patients with coronary care were excluded. This exclusion may affect the prediction of mortality.

## CONCLUSION

All three tested scoring models (APACHE II, SAP II and SOFA) would be accurate enough for our ICU patients. APACHE II has showed better calibration and discrimination power than SAP II and SOFA. Large further prospective validation studies of these predictive models should be conducted on large Pakistani ICU population before establishing a concrete conclusion.

## References

[1] Lewandowski K, Lewandowski M (2003). Scoring systems in the intensive care unit. Anaesthesist.

[2] Le Gall JR (2005). The use of severity scores in the intensive care unit. Intensive Care Med.

[3] Ridley S (1998). Severity of illness scoring systems and performance appraisal. Anaesthesia.

[4] Rothen HU, Takala J (2008). Can outcome prediction data change patient outcomes and organizational outcomes?. Curr Opin Crit Care.

[5] Woodhouse D, Berg M, van der Putten J, Houtepen J (2009). Will benchmarking ICUs improve outcome?. Curr Opin Crit Care.

[6] Kramer AA, Zimmerman JE (2007). Assessing the calibration of mortality benchmarks in critical care:The Hosmer-Lemeshow test revisited. Crit Care Med.

[7] Hosmer DW, Lemeshow S, Sturdivant RX (2013). Applied Logistic Regression, 3rd edition. John Wiley & Sons.

[8] Knaus WA, Draper EA, Wagner DP, Zimmerman JE (1985). APACHE II:a severity of disease classification system. Crit Care Med.

[9] Le Gall JRJ, Lemeshow SS, Saulnier FF (1993). A new Simplified Acute Physiology Score (SAPS II) based on a European/North American multicenter study. JAMA.

[10] Vincent JL, Moreno R (2010). Clinical review:scoring systems in the critically ill. Crit Care.

[11] Vincent JL, Moreno R, Takala J, Willats S, De Mendonça A, Bruining H (1996). The SOFA (Sepsis-related Organ Failure Assessment) score to describe organ dysfunction/failure. Intensive Care Med.

[12] Vincent JL, de Mendonça A, Cantraine F, Moreno R, Takala J, Suter PM (1998). Use of the SOFA score to assess the incidence of organ dysfunction/failure in intensive care units. Crit Care Med.

[13] Ferreira FLF, Bota DPD, Bross AA, Mélot CC, Vincent JLJ (2001). Serial evaluation of the SOFA score to predict outcome in critically ill patients. JAMA.

[14] Minne L, Abu-Hanna A, de Jonge E (2008). Evaluation of SOFA-based models for predicting mortality in the ICU:A systematic review. Crit Care.

[15] Pettilä V, Pettilä M, Sarna S, Voutilainen P, Takkunen O (2002). Comparison of multiple organ dysfunction scores in the prediction of hospital mortality in the critically ill. Crit Care Med.

[16] Knaus WA, Draper EA, Wagner DP, Zimmerman JE (1985). APACHE II:A severity of disease classification system. Crit Care Med.

[17] Le Gall J-R, Lemeshow S, Saulnier F (1993). A new simplified acute physiology score (SAPS II) based on a European/North American multicenter study. JAMA.

[18] Vincent JL, Moreno R, Takala J, Willatts S, De Mendonça A, Bruining H (1996). The SOFA (Sepsis-related Organ Failure Assessment) score to describe organ dysfunction/failure. On behalf of the Working Group on Sepsis-Related Problems of the European Society of Intensive Care Medicine. Intensive Care Med.

[19] Faruq MO, Mahmud MR, Begum T, Areef Ahsan ASM, Fatema K Ahmed (2013). A Comparison of Severity Systems APACHE II and SAPS II in Critically ill Patients. Bangladesh Crit Care J.

[20] Freire P, Romãozinho JM, Amaro P, Ferreira M, Sofia C (2010). Prognostic Scores in a Gastroenterology Intensive Care Unit. Rev Esp Enferm Dig.

[21] Haddadi A, Lademani M, Gainier M, Hubert H, Tange J, Micheaux PLD (2014). Comparing the APACHE II, SOFA, LOD, and SAPS II scores in patients who have developed a nosocomial infection. Bangladesh Crit Care J.

[22] Mbongo CU, Monedero P, Guillen-Grima F, Yepes MJ, Vives M, Echarri G (2009). Performance of SAPS3, compared with APACHEII and SOFA, to predict hospital mortality in a general ICU in Southern Europe. Euro J Anaesthesiol.

[23] Asadzandi M, Karati KT, Tadrisi SD, Ebadi A (2012). Estimation of the mortality rate using the APACHE II standard disease severity scoring system in intensive care unit patients. Iranian J Crit Care Nurs.

[24] Arabi Y, Haddad S, Goraj R, Al-Shimemeri A, Al-Malik S (2002). Assessment of performance of four mortality prediction systems in a Saudi Arabian intensive care unit. Crit Care.

[25] Semir N, Makhlouf B, Souheil E, Mondher J, Moez E, Rafik B (1997). Outcome prediction in intensive care:results of a prospective, multicentre, Portuguese study. Intensive Care Med.

[26] Moreno R, Morais P (1997). Outcome prediction in intensive care:results of a prospective, multicentre, Portuguese study. Intensive Care Med.

[27] Capuzzo M, Valpondi V, Sgarbi A, Bortolazzi S, Pavoni V, Gilli G (2000). Validation of severity scoring systems SAPS II and APACHE II in a single center population. Intensive Care Med.

[28] Katsaragakis S, Papadimitropoulos K, Antonakis P, Strergiopoulos S, Konstadoulakis MM, Androulakis G (2000). Comparison of acute physiology and chronic health evaluation II (APACHE II) and simplified acute physiology score II (SAPS II) scoring systems in a single Greek intensive care unit. Crit Care Med.

[29] Livingston BM, Mackenzie SJ, MacKirdy FN, Howie JC (2000). Should the pre-sedation Glasgow Coma Scale value be used when calculating acute physiology and chronic Health Evaluation scores for sedated patients? Scottish Intensive Care Society Audit Group. Crit Care Med.

[30] Tunnell RD, Millar BW, Smith GB (1998). The effect of lead time bias on severity of illness scoring, mortality prediction and standardised mortality ratio in intensive care—a pilot study. Anaesthesia.

